# Remarks on the Methods and Purposes of Pension Examinations1An address delivered before the National Association of United States Pension Examining Surgeons at its third annual meeting, Atlantic City, N. J., June 6, 1904.

**Published:** 1904-08

**Authors:** Sam Houston

**Affiliations:** Washington, D. C.; Medical Referee, Bureau of Pensions


### Remarks on the Methods and Purposes of Pension Examinations.[1]

By SAM HOUSTON, M. D., Washington, D. C.
Medical Referee, Bureau of Pensions.

AT YOUR annual session one year ago it was my pleasure and privilege to address you in the capital of our nation. Today, by your kindness and invitation, I have the appreciated honor of talking to you in "The City by the Sea," which for this week has become the Mecca of many learned medical societies; and, though your society is the junior of them all, it has discarded its swaddling clothes and today stands forth in the full vigor of youthful manhood. I am extremely sorry that it was not born forty years ago, and that our boards are not all represented here, thus to show greater interest in their work. However flattering the opportunity of appearing before you may be, I cannot but feel that a grave mistake was made in assigning to me the opening address. Possibly, however, this was not a mistake, as you will be better employed later in the session.

---

1. An address delivered before the National Association of United States Pension Examining Surgeons at its third annual meeting, Atlantic City, N. J., June 6, 1904.

I beg to assure you, at the outset, that the work of a majority of the examining boards during the past year has been of a higher order. The officers of the Bureau of Pensions feel that the boards of the country have been doing much better work and that they have been materially aided by this association. For myself, likewise, I can assure you that of very many of the boards I can ask no more than is being accomplished. I do not desire to attempt to inform you of the methods you should use in making your examinations, but to invite your attention to certain conditions not covered by the official book of instructions and which may be at times of great importance in determining a proper rate.

I know very well that your experience and education have prepared you to wrestle with the intricate problems of organic disease and the sequelæ as presented by objective symptoms, in accordance with the requirements of the pension law. I know of no body of physicians whose experience renders them more capable of detecting disease and sequences than the majority of our examining boards. They penetrate the almost hidden intricacies of disease and do it thoroughly, as exemplified by their work which is subjected to the scrutiny of a bureau of experts and by them approved. I trust you will pardon the egotism, when I explain that the experts of the central bureau are worthy of this distinction. These gentlemen each read and study many thousand certificates every year. The least error in diagnosis is detected and the sequelæ of the various diseases are as plain to them as the rotation of the seasons. With the certificates of medical examinations made by many of our examining boards and the fixing of rates thereon by the medical experts of the bureau, we feel satisfied that injustice is seldom done the claimant. So strongly am I impressed with many of our certificates of medical examination, that I believe that no *x*-ray machine yet invented can delineate the condition under examination better than the mental vision invoked by a perusal of the certificates.

One of the most difficult problems examining boards have to meet and solve is the differentiation of sequelæ and complications,—a necessary decision to be made under our pension laws where the original disease or disability is due to service, the sequential condition of disease proven by objective symptoms being equally pensionable with the primary disease. In this branch of medical knowledge members of examining boards and examiners in the bureau become expert physiologists and pathologists in detecting any departure from the normal function, or morbid anatomical change of organs or tissues.

It is true that a malingerer but very rarely appears before a board. But occasionally one will come, who has been coached by some unworthy member of our honorable profession who for revenue will thus prostitute his calling, and in this manner claimants for deafness, impaired vision, rheumatism, and even diabetes,—saccharine matter having been injected into the bladder,—have imposed upon us. Again, you are the recipients of numerous letters,—some from honest, honorable physicians, others from those who are not,—relative to the condition of a claimant who may appear before you for examination. The physician who is not honest, generally informs the claimant that he has a "pull" with the board of examiners and his influence will be all-powerful to bring the increase desired. If the claimant is successful, the physician with "influence" will claim he did it, and probably obtain a share. This is one of the most contemptible annoyances to which you are subjected, and if you should discover a physician engaged in this practice, we ask you to report him to the bureau. So far as your integrity has been surreptitiously assailed, we will see that no harm befalls you.

As you are well aware, the object in examining a claimant is to determine the degree of his physical and mental disability, and whether it renders him unable to perform manual labor wholly or in part. This is the basis upon which all pension enactments rest; the interpretation of the result, as reported by your certificates of examination, is our basis in the Bureau of Pensions by which to determine the rate. You have each received a book of instructions for your guidance in your examinations, but it only asks for results. All boards do not acquire these results in the same manner. Each board has its own peculiar way of making an examination, and we could not expect uniformity in manner without similarity in training, or more definite directions. We, however, do desire uniformity in the resulting description of the diseased conditions; not that the language shall be identical, but that it shall convey a clear conception of the disability claimed, so that the bureau can draw an accurate mental vision objective of the condition described. Theoretical speculations are not ordinarily desirable,—only the pathological conditions, based on objective symptoms are usually expected; and yet, there are times when we feel that we would be benefited by the impressions received by the board during the examination.

When, therefore, a claimant enters your examination room, observe him closely while he divests himself of his clothing. Is he erect or stooped? Does he walk with firm tread, or does he shuffle or hesitate in his locomotion? Does he hear your directions with regard to the disposal of his clothing? Is his mind clear, or are his answers incoherent? Does he manifest mental hebetude? By "sizing" up a claimant in this way you can give us briefly a first impression. Weigh and measure him; seat him and read his claims to him, entering others he may make; obtain his version of the length of time he has suffered from the affections named; note the circulation and temperature, as directed; obtain his exact age, if possible, and marks of identification. Then examine for disabilities claimed, underscoring those named in the order of the examination and paragraphing each disease or disability separately. Under the claim for gunshot or other wounds, please locate all such accurately on the diagram. Is the gunshot wound penetrating or perforating? If the former, was the missile removed, or does it remain? Describe accurately the entrance cicatrix. In perforating wounds, describe both entrance and exit cicatrices. In both cases, the probable course of a missile, although this is not easily determined, and the injury done to organs or tissues, may direct your examination for sequelæ.

The globular bullet, which inflicted many of the wounds during the civil war, was very erratic in its course. More modern conical bullets are hard to deflect from their line of flight. Saber and bayonet wounds are seldom seen.

The disease you are most frequently called upon to investigate is rheumatism, from 68 to 70 per cent. of all claims being founded upon this malady. While there are many and painful and exhausting subjective symptoms in this disease, we are only permitted to rate the condition on the objective symptoms, such as atrophy and hypertrophy of muscular and other tissues, and hardening of the white tissues with loss of function. It might be of benefit to us in fixing a rate to know something of the subjective symptoms. The pathology of rheumatism is not yet fully understood although, day by day, we are approaching the solution confirmatory, we believe, of the opinion that it is due to a want of oxidation of broken down excrementitious nitrogenous tissue, together with malassimilation of the digested food. The want of conversion of uric acid and salts which are sparingly soluble, into urea which is freely soluble and easily eliminated, permits the uric acid to accumulate in the white tissues of the body which are comparatively free of red blood and nourished by imbibition or osmosis. But this condition is only a sequence and is retroactive, the excess of uric acid in the blood being carried to all parts of the body admitting of the red blood's circulation, and thus it poisons the vasomotor nerve centers which control the circulation.

What influence the monoxide of carbon, a virulent poison, may have on precipitating an attack of rheumatism by poisoning the vasomotor and other nerve centers, has not as yet been determined. Carbon monoxide, which has a strong affinity for hemoglobin, suspends the oxygen-transporting function of the blood, and thus prevents the formation of urea from the nitrogenous waste of the tissues. Here, again, we find a want of carbonaceous oxidation into the negatively poisonous dioxide of carbon. This condition, commonly termed lithemia, may present no objective symptoms, yet the patient may suffer all the pains and pangs so fully and clearly described at our last meeting by Dr. Eisenberg and the gentlemen who engaged in the discussion of his paper. The intimate relation existing between the vasomotor and the sympathetic nervous systems, the latter presiding over nutrition, is interrupted and the harmonious action of these systems of nerves prohibited, producing hypertrophy in one case and atrophy in another, or both these conditions in the same individual in different stages of the disease; or, in still other cases no pathological condition may be apparent, only subjective symptoms being present. If this theorising is correct, we can see the futility and injustice of depending alone upon the objective symptoms.

The spinal theory of rheumatism advanced by Dr. J. K. Mitchell, 75 years ago, although he offered no pathological argument for his belief, has yet, I believe, some followers; and the microbe theory has an occasional adherent. The strongest argument for both these theories is that they can readily account for the metastasis of the disease. Comparative measurements for use in diagnosis can only be of value in cases of unilateral disease of this, or any other affection. In bilateral diseases they prove nothing beyond general atrophy or hypertrophy, as the case may present. Just here let me ask you not to decide that a claimant of local paralysis is a malingerer, simply because an electric current will cause contraction in a single or group of muscles when will-power will not be competent to produce action of the muscles. You may do the claimant an injustice by so deciding, since the natural stimulus is not as powerful in producing results as the applied current. Other and more reliable tests should be used.

Disease of the heart in those suffering from rheumatism is usually referred to as a result but, as you know, is independent of the rheumatic trouble manifested in other parts of the body. They are products frequently of the same cause. Both of these diseases may co-exist or only one may be found, depending upon the susceptibility of the involved organs to the uric acid poisoning. If one is found, it is well to carefully search for the other.

A frequent form of heart disease presented to us is dilatation and its sequences, and this is what we might expect. The youth of many of the soldiers of the civil war, the forced marches, the double quick and the excitement of battle were calculated to overtax the heart, and dilatation in many instances may have resulted. To carry out this suggestion a little further the dilated cavities have enlarged the openings until the valves have become too small, and regurgitation is induced, constituting valvular disease at some of the openings, generally the mitral. Frequently the youth of the soldier with nature's remedial power came to his assistance, and hypertrophy of the wall was induced patching up the heart to be almost as good as new, the only pathological condition remaining being an increase in size. This is the frequent history of heart disease in our civil war soldiers. Those in whom compensation never came to the rescue have either gone to their final reward, or are suffering a miserable existence. The "uric acid heart," and possibly the "monoxide of carbon heart" produce pathological conditions for which nature does not seem to have a remedy. The affections induced by functional disease of the heart, either pathological or physiological, are of import and should be noted. In this connection I desire you to carefully read the paper presented to the association a year ago by Dr. Frost, and the discussion following it.

Perhaps there is no pensionable disease that occasionally requires such close investigation as that of chronic diarrhea. The simple ordinary form depending upon looseness of bowels and offensive discharges, producing sunken abdomen, despondent expression, irritability of bowels, gradual and continuous emaciation, is easy to diagnosticate; but that form which assumes the intermittent, ulcerative type and is associated with malaria, in which the pathological condition is confined to the lower colon and consists in periodical ulceration with exacerbations, the upper intestinal tract being comparatively free from disease under a proper dietary, there being no wasting of the body—what is lost during the periodical attacks being regained in the intermission,—is difficult to diagnosticate, seeing claimants only during the intermission as you do, and it is equally hard to give a proper rate, especially when accompanied by those almost constant attending sequences,—hemorrhoids, proctitis, and prolapsus, the result of weakened rectal tissues and frequent tenesmus. These claimants are in great danger of being underrated, since they are able to attend to some light business a portion of their time, but at a fearful cost of pain and suffering, always in danger of colonic perforation or malignant disease of the parts involved.

Another disease quite often met with in old soldiers is naso-pharyngeal catarrh. The objective symptoms are so apparent, and you are so conversant with them, that I only desire to call your attention to the fact that when in these cases you find ulceration to admonish you to examine the case exhaustively, feeling assured that you will find a history of syphilis or some other constitutional dyscrasia. As has been well remarked by Dr. Potter, in his address at our last meeting, catarrh does not change its type to become tubercular. If tuberculosis is found, it is a complication, and not a sequence.

The chronic bronchitis of soldiers is found generally in lithemic individuals. Some authorities believe it is a sequence of rheumatism. This disease may continue for years with slight disturbance to general health, but it may simulate phthisis. Cough and expectoration with emaciation will be present and mark the soldier as a proper recipient of the nation's bounty. A proper differentiation of asthma is desired in all certificates of examination. It is very important that we should know whether it is the neurotic or cardiac type, the latter being a much more formidable disease than the former.

I desire to thank the association for the very valuable medical papers read to the association a year ago, and which have been so nicely printed and bound and presented to our examining boards and the Pension Bureau. These papers are an honor to the association and of value to every physician. We hope that the examining surgeons will all aid in this good work.

In conclusion permit me to say that the commissioner of pensions and his subordinates in the pension office desire that every claimant should have a thorough examination and be fully rated under the law for all pensionable disabilities. In accomplishing this, we ask your hearty coöperation. We also ask for the old soldiers your kindly consideration during their examination.

